# Nanotechnology as a Key to Enhance the Benefits and Improve the Bioavailability of Flavonoids in the Food Industry

**DOI:** 10.3390/foods10112701

**Published:** 2021-11-05

**Authors:** Jocelyn C. Ayala-Fuentes, Rocio Alejandra Chavez-Santoscoy

**Affiliations:** Tecnológico de Monterrey, Escuela de Ingeniería y Ciencias, Av. Eugenio Garza Sada 2501 Sur, Monterrey 64849, Mexico; A01329401@itesm.com

**Keywords:** nanoencapsulation, bioavailability, flavonoids, biopolymers

## Abstract

Nanotechnology has impacted the food industry, mainly on developing healthier, safer, and high-quality functional food. Flavonoids are valuable compounds present in plants, fruits, grains, roots, stems, tea, and wine, among others; they possess many benefits for health due to their antioxidant properties toward reactive oxygen species, anti-inflammatory, and antiproliferative, among others. These characteristics make flavonoids attractive in various industrial areas such as medicine, nutraceutical, cosmetology, and pharmaceutical. Unfortunately, flavonoids lack long-term stability, are sensitive to light, long periods of darkness with low oxygen concentration, and often present a low water solubility and poor bioavailability. Nanoencapsulation is an alternative to improve bioavailability and sensitivity in the manufacturing process, based on encapsulating substances on a nanoscale. Nanocapsules are a promising strategy in significantly enhancing the delivery of compounds to various sites in the body. The development of biopolymers to encapsulate sensitive compounds is increasing, as well as the search for the non-toxic, biodegradable, natural and biocompatible characteristics of polymers, is fundamental. The present review describes the recent techniques and technologies for the nanoencapsulation of flavonoids. It discusses their potential advantages and possible limitations, compares natural and synthetic biopolymers, and finally, details nanoparticle regulation.

## 1. Introduction

Nowadays, the consumer demands quality and health benefits in their food while preserving all the organoleptic properties. The importance of conserving food quality and health benefits without disturbing nutritional value in industrial processes has been a challenge.

The development of nanotechnology has supported transforming numerous areas of food science and the food industry with increasing investment. In recent decades, nanoscale engineering and manufacturing have made it possible to improve different processes in various areas, such as chemical, physical, biological, electronic, and engineering sciences [1].

The food industry has increased demand for nanotechnology applications; it provides strategies to solve significant problems ranging from food manufacturing and processing to packaging. Especially in food processing, nanomaterials can be used as food additives to improve sensory properties, transport nutrients, antibacterial agents, etc. [2]. According to its functions, the application of nanotechnology in food can affect the bioavailability and nutritional value of food. Nevertheless, the biological properties of nanomaterials still depend on their physicochemical parameters [3]. In addition, food nanotechnology can enhance taste, texture, and consistency. It also helps suppress the unpleasant taste or odor and modifies the particle size, size distribution, possible cluster formation, and surface charge [4].

Incorporating bioactive compounds into food is challenging because they tend to have very low chemical stability. Thus, absorption in the human body is generally limited. In addition, bioactive compounds tend to have low solubility in water or low bioavailability [5]. These are some of the reasons that do not allow consumers to experience their benefits. Bioavailability means that a substance or drug becomes entirely available for its intended biological destination(s) [6]. Nutraceuticals exhibit different hydrophilic and hydrophobic properties, affecting their assimilation into the human body.

Meanwhile, bioaccessibility refers to the portion of the compound that is released from the food matrix and is available for absorption [5]. Bioaccessibility for hydrophobic compounds includes how these molecules are incorporated into micelles in the small intestine to be absorbed in the gastrointestinal tract (GIT), similar to lipids.

Compounds are absorbed through the epithelial layer of the intestine mainly by two mechanisms: passive transport and active transport. The characteristics of bioactive compounds determine the mechanism of absorption, and bioaccessibility and bioavailability are two phenomena that influence the absorption process [5].

The unique properties of nanomaterials provide significant advantages for food processing. They are mainly used as ingredients or supplements [7]. Nanoparticles have better properties for encapsulation, and they release bioactive compounds with more efficiency than traditional encapsulation systems. In addition, particle size directly affects the delivery of compounds to numerous tissues in the organism. Interestingly, several reports suggest that only nanoparticles can be efficiently absorbed into specific cell lines, as opposed to larger microparticles, which significantly reduce their absorption [6]. In addition, nanomaterials could improve compatibility with the food matrix, increasing the shelf life and bioavailability of the food products [8]. However, the toxicity and safety of nanomaterials have not been thoroughly studied and documented [9]. However, there are agencies in charge to legislate and regulate nanomaterials, such as the United States Food and Drug Administration (U.S. FDA) and European Commission (EC). Several nanometric delivery systems have been designed and approved for the food, supplements, and pharmaceutical industries. The primary purpose of nanometric delivery systems is to improve the digestion, release, and absorption of lipophilic bioactive compounds, or chemically unstable molecules. Bioactive compounds are essential for physiological functions in the human body. Additionally, many reports describe host disease prevention by the consumption of nutraceuticals [5].

Nanotechnology also plays an essential role in reducing food waste due to food processing or spoilage. Nanotechnology could help increase the shelf life of food and improve the containment and preservation of food in food packaging [10]. However, nanostructured food ingredients and delivery systems for nutrients and supplements comprise the main focus in nanotechnology applications in foods [11].

## 2. Flavonoids

Flavonoids are phenolic substances isolated from natural sources because they belong to a class of plant secondary metabolites. They are the most common phenolic compounds in a flowering plant, and they are found in the human and animal diet. The basic structure is the flavan nucleus, it consists in three rings (C6(A)-C3(B)-C6(C)). The classification of flavonoids depends on the position of linkage of ring B, and their classes are anthocyanins, chalcones, flavones, flavanones, flavanols, and isoflavonoids [12].

The interest in flavonoids for the food industry and food science is because they have a wide range of biological activities. The most-reported bioactive activities of flavonoids are antioxidant, antitumor, anti-inflammatory, hepatoprotective, lipid and carbohydrate metabolism regulators, and antiviral properties. They have gained increasing attention in therapeutic effects in different diseases such as chronic disorders including metabolic, neurodegenerative diseases (Alzheimer), cancer, autoimmune illness, cardiovascular disorders [12], and recently, as a possible potential antiviral against COVID-19 [13].

The bioavailability of flavonoids is generally low and may vary drastically among different flavonoid classes and individual compounds in a particular group [14]. Efforts are being made to improve the bioavailability of flavonoids. Genetic engineering is a recent tool to enhance plant performance and generate other sources of production such as microorganisms. However, microbial production of flavonoids has been achieved only at a laboratory scale, maybe due to obstacles such as adding expensive precursor substances [15]. Thus, the industry focuses on new ways to take advantage of the limited extraction of flavonoids and their maximum use in the human body through stabilizing the compounds to preserve their properties until reaching the mechanism objective.

**Figure 1:**
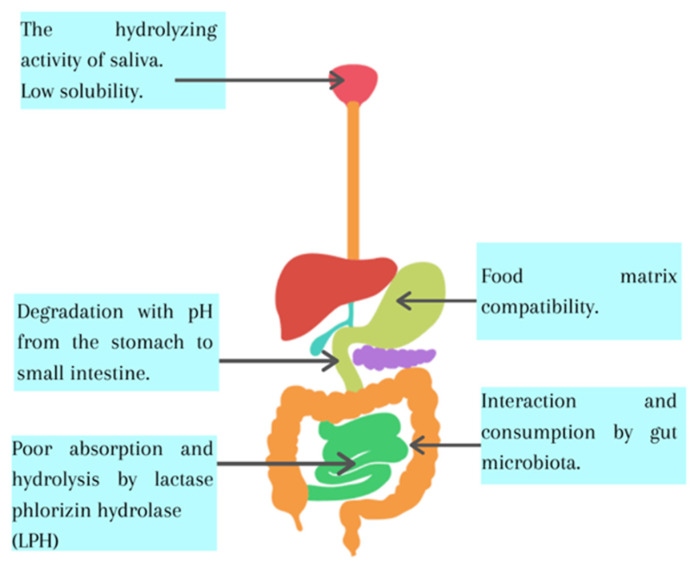
Summary of the factors accounting for the low bioavailability of flavonoids.

The main route of absorption of flavonoids is the gastrointestinal system because the primary sources of flavonoids are plants, fruits and derivatives. Flavonoids have low bioavailability, which has been associated with their degradation at various stages of the digestion process, including degradation due to the activity of gastrointestinal hydrolytic enzymes, their low distribution of the food matrix to the lumen of the intestine, the composition of the microbiota and its inefficient absorption by intestinal epithelial cells [14,16]. When ingesting flavonoids, the first stage is chewing, and then in gastric phase, the main part of food mass is mainly hydrolyzed due to acid pH. As digestion passes from the stomach to the small intestine, the pH gradually increases in the small intestine from a pH of 6 to about a pH of 7.4 in the terminal ileum, and the pancreas and bile secrete various enzymes and biosurfactants, All of these factors change the structure of flavonoids. Finally, in the colon, the gut microbiota uses the benefits of flavonoids in a different form and their derivates. The reasons for the low bioavailability of flavonoids are briefly summarized in Figure 1.

The structure of flavonoids, food matrix, processing, and external conditions may interfere in the bioaccessibility of flavonoids [17]. As mentioned before, bioaccessibility has defined the portion of a food constituent liberated from a food matrix in the gastrointestinal tract and become available for absorption [18]. The food matrix is the most critical factor that affects bioavailability and absorption in the consumption of flavonoids. Thus, it is necessary to consider the interaction of flavonoids with other components in the food, such as proteins, fibers, vitamins, etc. [17]. Processing and external conditions are two factors that go hand in hand; both are important on the laboratory and industrial scale because they directly influence the structures and, consequently, their biological activities. There is evidence that the storage conditions can be a limiting step affecting product quality. Chaaban et al. [19] studied six different flavonoids and their stability in two variables in storage conditions, light and oxygen. They observed that flavonoids are not stable in the dark with a low quantity of oxygen. Additionally, the flavonoids without the hydroxyl group in the third position show the highest stability compared to flavonoids that have it [19]. Besides, refrigeration, heat, environment, food industry processing and domestic processing, and domestic process showed degradation of flavonoid content [16].

## 3. Nanoencapsulation

Nanoencapsulation has been commonly used to preserve and improve the benefits of unstable or sensitive compounds. It is the process of entrapping one substance in another, producing particles with diameters on a nanometric scale [20]. There are two forms to apply encapsulation in the food industry, microencapsulation, and nanoencapsulation. First, microencapsulation refers to capsules with a size range of 1 to 1000 μm, which are small particles of solid, liquid or gas within a secondary material. In contrast, nanoencapsulation refers to nanoscale encapsulation; bioactive compounds are loaded into capsules surrounded by a variety of materials that protect them.

The wall materials, during micro and nanoencapsulation, can protect flavonoids (as a shield) from different factors that could decrease their bioavailability and bioaccessibility in the human body [21,22]. Micro and nanoencapsulation are applied in the pharmaceutical, agricultural, cosmetic, medical, and food industries to protect bioactive compounds. However, nanoencapsulation technologies provide possible solutions to inherent difficulties associated with macro and microscale encapsulation to deliver bioactive compounds.

Nanocapsules allow overcoming compatibility issues with the food matrix, such as aggregation and phase separation, which frequently affect the appearance of the final product, texture, flavor, stability, and color. Additionally, nanoencapsulation allows us to design the controlled and intelligent delivery of bioactive compounds. Intelligent delivery of bioactive compounds refers to releasing the compounds under specific conditions that enable their release into target tissues [23]. Therefore, nanoencapsulation allows us to overcome the challenges that previously avoided taking advantage of the functional and medicinal properties of bioactive compounds. Finally, nanoscale delivery systems must be biocompatible for the human body and economical for the food industry and other industries.

### 3.1. Nanoencapsulation Technologies

**Figure 2:**
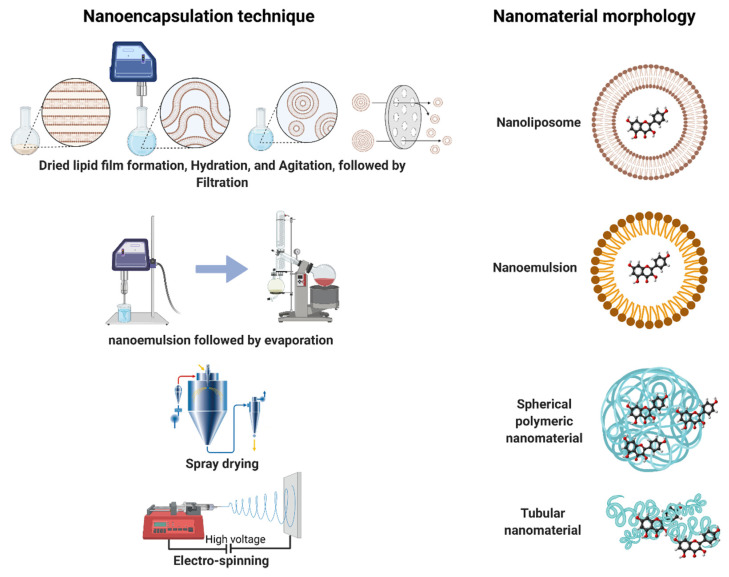
Most used techniques for nanoencapsulation of flavonoids.

Recently, the demand for new products with healthy and beneficial compounds has increased over recent years in different industries. That is the food industry and nutraceutical case with the trend to consume “*functional foods*”, which are designed to improve human health, well-being, and performance [24]. Thus, different techniques for nanoencapsulation processes were developed. Figure 2 shows the most used methods to nanoencapsulate flavonoids.

The selection of the best method depends on several parameters, such as the nature of the encapsulant, food formulation, food processing, the final application, and the cost of the process [22]. Table 1 summarizes the advantages and limitations of nanoencapsulation methods applied for flavonoids.

#### 3.1.1. Nanoemulsion

Nanoemulsions are liquid-in-liquid dispersions with sizes from 1 to 100 nm; they are formed by mixing oil, emulsifier, and water, followed by evaporating the continuous phase [25]. They are very versatile because they can be simply produced using natural food ingredients. Nanoemulsions may be designed to enhance water dispersion and bioavailability [8]. Nanoemulsions present high stability under moderate conditions, such as pH value, temperature, or salt concentration [26]. Moreover, their small droplet dimensions exhibit benefits such as high optical clearness, excellent physical constancy against gravitational partition, and droplet accumulation, making them suitable for food applications [27]. According to Singh et al. the term nanoemulsion is sometimes confused with microemulsion, but both differ in structural aspect and long-term thermodynamic stability [28].

The increase in bioavailability is due to the nature of nanoemulsions. They have two phases: the oil phase and the water phase stabilized by an interfacial film of surfactant. It improves nanoparticles solubility in the gastrointestinal fluid. Moreover, nanoemulsions might enhance the permeability through the intestinal wall and protecting bioactive from enzymatic degradation in the GIT. These formulations can successfully improve the undesirable oral absorption of the poor water-soluble flavonoid compounds [26].

**Figure 3:**
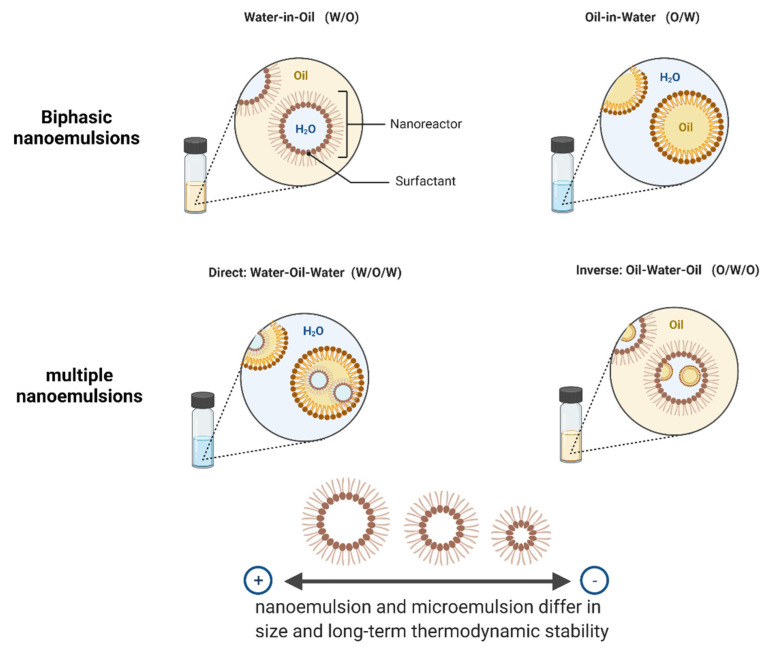
Classification of nanoemulsions.

Nanoemulsions can be biphasic or multiple, the difference is in the nature of the dispersed phase and the continuous phase. These can be classified as biphasic oil-in-water (O/W) and water-in-oil (W/O) nanoemulsions and multiple nanoemulsions water-oil-water (W/O/W) or oil-water-oil (O/W/O) [28], they are usually named direct and inverse nanoemulsions, respectively (Figure 3). Nanoemulsions are typically prepared by either low-energy or high-energy methods or a combination of both. The low-energy methods involve spontaneous emulsification. High-energy methods require using mechanical devices, such as high-pressure homogenizers, microfluidizers, or ultrasonic homogenizers to generate intense, disruptive forces to produce emulsification and/or reduce the particle size. The main advantages of using methods that require low energy are their simplicity, speed, and low cost. In contrast, the use of high-energy methods to make nanoemulsions require low cosolvents (e.g., propylene glycol, glycerol, and sorbitol) or cosurfactants (e.g., short and medium-chain alcohols) and a variety of ingredients can be used [25,26].

According to Chen and Inbaraj [25], anthocyanins are water-soluble flavonoids that might enhance their physicochemical stability in nanoemulsions with different oil, water, surfactant, and cosurfactant ratios for topical skin application and urinary tract infection. The formulated W/O anthocyanin nanoemulsions are stable in storage conditions after 30 days. They showed no phase separation, and samples exhibited antioxidant activity, high retention rates of polyphenols, and a half-life of 385 days [29].

Although nanoemulsions represent a method adequate to soluble flavonoids, there are efforts to modify and improve the technique for insoluble compounds, as quercetin. Quercetin is a lipophilic compound with many reported therapeutic effects. Multiple studies have shown that quercetin solubilizes in a differentiated manner depending on the matrix that contains it. For example, quercetin solubilizes closer to polar groups in lipid membranes, but has intermediate polarity between hydrophobic and hydrophilic molecules [30]. Chen et al. [31] developed quercetin-loaded rice bran protein-based nanoemulsion with satisfactory results. The inclusion rate was 98.12 ± 0.07%, and the droplet size of 219.7 ± 2.1 nm. Quercetin nanoemulsions had higher stability at alkaline conditions and low salt concentrations, which simulate oral administration. Therefore, rice bran protein-stabilized nanoemulsions were considered efficient carriers for transporting and promoting the bioavailability of insoluble and sensitive molecules within the GIT.

#### 3.1.2. Spray Drying

Spray drying is the oldest and most widely used technique for microencapsulation, and now, nanoencapsulation. It is used in the food industry due to its flexibility as well as being more economical. This method does not affect the sensory and textural characteristics of a finished product [32]. The biggest challenge in using spray drying is to achieve a high product yield with maximum encapsulation efficiency. The principal factors that affect the product yield and encapsulation efficiency are the selection of the feed formulation (solids content, surface tension, viscosity, and the concentration of wall and core materials) and operating parameters (inlet and outlet temperatures, drying gas flow rate, feed flow rate, and atomizer pressure and speed) [33]. Additionally, the atomization system is the part of the instrument with a significant influence on the size of particles. The particle size obtained by conventional spray drying usually varies from small size (1–5 μm), medium size (5–25 μm), and large size (10–60 μm) powders. Interestingly, the appropriate manipulation of the atomization conditions contributes to the final size [34]. The main advantage of spray drying is that particle formation occurs in a single step. Additionally, it is a continuous and scalable process.

It is necessary to make an emulsion to generate nanogels; then, it is sprinkled into small droplets, and finally the solvent evaporates, resulting in small droplets of the product stored in the carrier substance and embedded in the nanogel [35]. It is also possible to obtain a liquid product embedded inside a solid matrix, leaving a solid matrix around the dispersed second phase. In this case, the feed formulation must be a multiple emulsion. Multiple emulsions contain, as a dispersed phase, an inverse emulsion, and the continuous phase is an aqueous liquid. They are water-in-oil-in-water (W/O/W) emulsions. This type of emulsion is basically used in pharmacy, as it allows obtaining a prolonged release of the drugs.

Pedrozo et al. [36], produced bovine serum albumin-based nanoparticles, which contain rutin by nano-spray drying. Rutin corresponds to flavonols classification of flavonoids, which is a hydrophobic molecule. The result showed a low encapsulation efficiency of around 32%. It may be due to the following factors: the low affinity of bovine serum albumin to rutin, or the degradation of rutin during spray drying due to the high temperatures involved in the process. Although high temperatures limit spray drying when using heat-sensitive compounds, there are advantages compared to other methods. The degradation during storage conditions is normally lower in comparison with freeze-drying and follows first-order kinetics. Besides, as a wall material for flavonoids are biopolymers, and sometimes there is no need to apply emulsifiers during spray drying due to hydrophilic behavior. The encapsulating materials are generally carbohydrates with different complexity and length. The most common are alginate, gums, starch, chitosan, cellulose derivatives, and maltodextrin. There are other common encapsulating materials of different nature, such as gelatins, proteins, and smaller peptides [31].

#### 3.1.3. Electro-Spinning

Electro-spinning is an innovative technique for the production of polymeric nanofibers; the electrostatic forces cause nanofibers formation from the electrically charged jet of a polymer solution followed by depositing onto a grounded collector. Additionally, several parameters can influence the production of nanofibers by electro-spinning, for example, the solution properties, such as viscosity, conductivity, concentration and molecular weight of the polymer, viscosity, etc. The processing conditions also affect the characteristics of final nanofibers. Some of them are the hydrostatic pressure in the capillary tube, the distance between the tip and the collecting screen, the volume and rate of feed solution, and the electric potential at the capillary tip. Additionally, ambient parameters can also affect the process, e.g., feed solution temperature, humidity in the electro-spinning chamber, air velocity, among others [37].

This method offers an alternative to stabilize flavonoids with a structure that are thermodynamic unstable. Additionally, it can enhance the poor slow-release performance obtained in the nanoemulsion technique. Zhan et al. [38] developed nanofibers by electro-spinning. They encapsulated tangerine as a water-insoluble drug delivery made of water-resistant poly (vinyl alcohol)/poly (acrylic acid). Tangerine is a flavonoid with more than two methoxy groups on the chemical skeleton (flavones class). It has beneficial effects such as antioxidant, antitumor, anti-inflammatory activity, etc., but its bioavailability is limited due to its poor solubility and high melting point. The results showed that methodology and polymer do not affect antioxidant activity. Other successful cases are the use of β-cyclodextrin as the host and quercetin to generate nanofilm by electro-spinning. These nanofibers exhibited quite high antioxidant activity and photostability. Furthermore, they had an efficient inhibitory effect on *E. coli* and *S. aureus* and prolonged the active time of quercetin [39].

Few studies have been focused on the encapsulation of phenolics for the food industry through electro-spinning techniques. The main limitation of applying these techniques in the food industry is the final cost of the product, which is higher compared to other technologies. Other reasons are the low production yield and the lack of reproducibility of food-grade nanofibers. The scaling process usually becomes challenging, along with the high investment required and the challenges in working with biopolymers. Consequently, the main application of these techniques is still in the pharmaceutical field for drug delivery systems [40].

#### 3.1.4. Nano Liposome

Liposomes are defined as colloidal spherical structures surrounded by polar lipids. In these colloidal spherical structures, the arrangement of lipophilic tails away from the water is toward the center of the vesicle, while the orientation of hydrophilic heads is toward the aqueous phase. Nanoliposomes involve the preparation of conventional liposomes and the reduction of the particle size using high-pressure homogenization, membrane extrusion, or ultrasound [25]. Phospholipids mainly produce liposomes as bilayer components. Usually, phospholipids are obtained from many food resources, such as soy, pea, egg, and milk [22].

Several disadvantages have been related to conventional nanoliposome preparation methods: heterogeneous size distribution, high energy cost with the multi-step operation, low encapsulation efficiency and reproducibility, lack of long-term stability, and the presence of solvent/surfactant residue are some of them. Improved methods could overcome these limitations. Those improved methods are freeze-drying double emulsion, microfluidic hydrodynamic focusing, dual asymmetric centrifugation, crossflow filtration detergent depletion, membrane contactor technology, and the use of supercritical CO_2_ technology [25].

Hao et al. [41] produced chitosan-coated nanoliposomes delivery carriers of quercetin. These nanoliposomes showed an encapsulation efficiency of 71.14%, and increased the stability of quercetin in storage conditions at 4 °C and 25 °C in natural light. Quercetin was successfully encapsulated in chitosan coating by the electrostatic deposition method, utilizing the electrostatic interactions between positively charged chitosan and negatively charged phosphates. Furthermore, it has been reported that nano-encapsulated quercetin exhibited similar cytotoxicity compared to free quercetin on HepG2 cells. Thus, nanoencapsulation systems based on nanoliposomes have enormous potential to be applied in the food industry to conserve the quality and benefits of quercetin.

The development of new strategies to generate safe and straightforward nanoencapsulation methods has increased, causing an area of opportunity for the nanotechnological field. Nanoliposomes are the case. Sun et al. [42] developed a simpler process using the ethanol injection method to combine ultrasonication to generate anthocyanins-loaded liposomes. It also has many advantages, including the higher encapsulation efficiency (91.1 ± 1.7%), the smaller particle size, and the higher absorption. Nanocapsules proved a negative influence on the proliferation of cancer cells (Caco-2 cells). This effect was due to anthocyanins and the phospholipids (lecithin) used in the preparation of nanoliposomes.

Recently, the trend of using biopolymers, for coating liposome droplets with polymers, to improve liposome stability has increased. The results in the use of chitosan and lecithin showed that these liposomes could be used as an appropriate delivery system for functional bioactive compounds. Table 2 shows the most applied methods for particular flavonoids encapsulation.

### 3.2. Biopolymers

One of the major obstacles in food applications is the replacement of nonfood-grade materials by bio-based, biodegradable food-grade alternatives. Currently, many research groups are interested in using biodegradable polymeric nanoparticles in food and pharmaceutical fields due to their promising properties of flavonoids such as good biocompatibility, accessible design and preparation, structure variations, and interesting bio-mimetic characters [35].

The external polymeric membrane is essential in nanocapsules synthesis. The selection of polymer according to its physicochemical properties plays a critical role in the responsiveness of the nanomaterial. Therefore, a few factors must be considered before deciding on a polymer. These include whether the polymer and its degraded products are safe, non-toxic, non-immunogenic, and biodegradable, or eliminated from the body in a short period of time [49]. The nanoparticles could be made from natural or synthetic polymers, and they also could be biodegradable or non-biodegradable. Polymers can be customized for targeted delivery of compounds, provide a controlled or prolongated release of compounds, improve bioavailability, and/or prevent endogenous enzymes from degrading the bioactive compound [50].

The biodegradable biopolymers are divided into natural and synthetic polymers. The commonly natural biopolymers used to prepare nanoencapsulated materials are chitosan, gelatin, sodium alginate, and albumin [41]. Natural biopolymers can be generated from proteins and polysaccharides. The most employed proteins and carbohydrates are gelatin, soy and milk proteins, and chitosan, alginate, and starch. On the other hand, the most utilized synthetic polymers are poly(lactic acid), poly(d,l-glycolide), poly(lactide-co-glycolide), among others (Table 2) [35]. However, general challenges persist in applying biodegradable nanoparticles, circulation time, drug incorporation, and efficiency because nanomaterials compete with the degradation rate of the bioactive compounds [51]. Table 3 presents the main advantages and limitations of the most used polymers for flavonoid nanoencapsulation.

The hybrid nanoparticles show advantages compared to nanoparticles made of one material. They endow multifunction to the delivery system and are usually coated with polysaccharides [52]. Different combinations of polysaccharides (polysaccharides with protein, lipid, synthetic biopolymer) have an advantageous effect on flavor retention and release characteristics [53].

In some cases, the performance of biopolymers is related to the method and technology chosen to produce nanocapsules. It is the case of electro-spinning, where biopolymers have proven to be quite challenging to form gels through hydrogen bonding. To counteract this limitation, adjusting an optimal concentration is critical, as it determines the morphology of the final product [38]. On the other hand, some studies have shown that combined biopolymers result in better efficiency and stability in the encapsulation process in the spray drying method. Still, other studies contradict this statement, ensuring that a combination of two or three biopolymers can decrease the encapsulation efficiency [54,55]. Consequently, the choice of nano-carrier should be controlled by the nature of the bioactive compound, the presence of surface groups, temperature and pH during processing, release rate, and degree of cellular uptake, as well as economic considerations [21].

Due to the application of biodegradable materials, the toxicity of nanomaterials has mainly been decreased, but not all biodegradable materials are considered safe for human consumption. Despite their biodegradability, some nanoparticles may still have unwanted side effects, for example, on the blood coagulation system due to their physicochemical properties [43].

## 4. Safety and Regulatory Issues

Nowadays, the possibility of humans spreading and being exposed to nanoparticles is rapidly growing. This justifies a thorough study and review of the advantages of nanoparticles as alternatives to improve the bioactive and sensory properties of products, knowledge about their potential toxicological and side effects, and the impacts of the use of nanomaterials on the environment [30]. Nevertheless, few studies have focused on the potential toxicity or environmental impact of nanomaterials in foods [3]. Countries worldwide have developed an organization that regulates nanotechnology, especially the European Union (EU) and US food and Drug Administration (FDA). FDA has formed an internal FDA nanotechnology task for determining regulatory approaches for nanomaterials [56]. In EU, food with nanomaterials must be notified to consumers [57]. Ensuring consumer confidence and acceptance of food containing nanomaterials remain a challenge for the government and industry.

Further studies are needed to overcome the limitations of current nanoencapsulation processes methods and their production at an industrial scale. Additionally, there is a need to generate information about the optimization of formulations and encapsulation systems, as well as to meet commercial demands and to explore the application of nanosized food ingredients characteristics and gastrointestinal systems [21]. It is necessary to documented the impact of nanoparticles on the human body (i.e., nanotoxicity), and the influence of particle size, mass, chemical composition, surface properties, and the aggregation of individual nanoparticles in their toxicity [58].

Furthermore, the use of GRAS substance (generally regarded as safe) as a nanomaterial does not guarantee safety. Other studies must be developed to analyze the potential hazards of nanoparticles in food products. This is because the physicochemical properties at the nanoscale are entirely different from those found at the macro-scale. Besides, the size of these nanomaterials may increase the risk for bioaccumulation within body organs and tissues [59].Therefore, it is essential to consider the following aspects to reduce the possible side effects of oral bioavailability:

- Discover effective and non-toxic materials obtained from natural products for the production of nanomaterials used as absorption enhancers.
- Systematically evaluate the potential toxicity of nanoformulations in vitro and preclinical study.
- Determine in detail the mechanism of action through which the nanomaterial exerts its effect, for example, the absorption enhancer to adequately control its concentration and exposure time in intestinal epithelial cells [12].

Finally, and as mentioned above, there is not enough scientific information concerning the impacts of nanotechnology on the environment and ecosystem. Besides, nanosized food ingredients and nano packaging materials might impact general human health and the immune system. Consequently, if there is increasing effort in the design and development of nanomaterials, it is necessary to constantly study their side effects and toxicity throughout in vitro assays, animal studies, and even clinical trials for long-term follow-up [58].

## 5. Conclusions and Future Perspectives

Flavonoids are compounds with several benefits to human health. They are present in many vegetables, fruits, plants, and food, but the human body cannot take advantage of all properties due to poor bioavailability during GIT. Furthermore, they represent a challenge in the food industry because of their sensitivity in manufacturing and storage conditions. However, the food nanotechnologies open several possibilities to improve the stability and benefits of flavonoids according to structure, storage conditions, processing, and final application.

This review summarizes the role of nanotechnology in food science; in specific, food processing focuses on improving the bioavailability of flavonoids and discusses some facts associated with the safety and regulation of this technology. The nanoscale improves bioavailability in the gastrointestinal tract. Several technologies have been used to stabilize bioactive compounds. Nanoemulsions, nanoliposomes, spray drying, and electro-spinning are the most used techniques to stabilize flavonoids. Each one has advantages and disadvantages depending on the desire application and the properties of the used flavonoid. Additionally, there is a tendency to develop nanocapsules elaborate with biopolymers. Biopolymers are divided into two categories, synthetic and natural. Both have a lot of benefits, such as non-toxic, biocompatible, biodegradable, etc.

Although nanotechnology is an alternative to improve the bioavailability of flavonoids, security and regulations are evolving slowly. Regulatory institutions already exist, but there is still a gap in long-term health and environmental impact research. As an area of opportunity, more studies are needed to develop technologies that generate low-cost nanoparticles that improve the bioavailability of flavonoids in the food industry.

## Figures and Tables

**Table 1:** Nanoencapsulation methods applied for flavonoids.

Technique	Morphology	Advantages	Disadvantages
Nanoemulsion	Vesicles/spherical	Encapsulate high concentrations. Low energy.	High cost. Unstable during log-tern storage.
Nanoliposome	Bilayer lipid vesicles	Stable against environmental factors.	Difficult scale-up. Sensitivity to mechanical stress.
Spray drying	Spherical/compact	Industrial-scale. Less processing time. Hydrophobic compounds without emulsifiers. Low operating costs.	High temperature. Retention of a compound is generally lower.
Electro-spinning	Tubular	Absence of heat. High surface retention. High encapsulation yield and porosity.	Limitation in industrial scale. Challenge in setting parameters.

**Table 2:** Nanoencapsulation techniques of various flavonoids.

Flavonoid(s)	Excipient	Technique	EE (Encapsulation Efficiency) %	Reference
Rutin	BSA (Bovine serum albumin)	Spray Drying	32%	[36]
Quercetin	Casein and casein with 2-HbetaC	Spray Drying	Without 2-HbetaC: 75.4% With 2-HbetaC: 82.9%	[43]
(−) Epicatechin	Lecithin-chitosan	Nanoemulsion	56%	[44]
Catechins	Zein	Electro spraying	>85%	[45]
Hesperetin	Polyamide (PA)	Nanoliposomes	98.1%	[46]
Fisetin	Poly-(ε-caprolactone) (PCL) and PLGA-PEG-COOH (poly(d,l-lactic-co-glycolic acid)-block-poly(ethylene glycol) carboxylic acid)	Nanoprecipitation	70–82%	[47]
Anthocyanis	lecithin	Nanoliposome	85.60%	[48]

**Table 3:** Advantages and limitations of biopolymers.

Biopolymers	Example	Advantages	Limitations
General Biodegradable Nanoparticles	Formulated using FDA-approved	- Biocompatibility. - Low immunogenicity. - Targeted delivery. - Enhanced therapeutic effects. - Modifiable size and surface.	- Unstableif not modified. - High-cost production. - Heterogeneous size and structure. - Low encapsulation efficiency. - Difficulty in large scale production. - Difficulty in cleaning or sterilization.
Synthetic	PLGA (D, L-lactic- co-glycolic acid) PLA (lactic acid) PLG (D, L-lactide-co-glycolide)	- Long drug release period. - Mechanical and chemical stability. - Reproducibility. - Nonspecific protein binding. - Ease of modification. - Tunable properties.	- Possibility of toxic and non-degradable. - Complex and costly production process.
Natural	Chitosan BSA (Bovine Serum Albumin) Sodium alginate	- The high amount of hydroxyl groups on their backbone. - Biodegradable (degraded into components that are easily re-absorbed or eliminated). - Biocompatible. - Adhesion or attraction to target tissues. - Increased residence time. - Neutral coating with low surface energy.	- Variability in animal sources. - Complex structures. - Complicated and costly extraction processes.

## Data Availability

Not applicable.
